# Reciprocal interactions of mouse bone marrow-derived mesenchymal stem cells and BV2 microglia after lipopolysaccharide stimulation

**DOI:** 10.1186/scrt160

**Published:** 2013-01-28

**Authors:** Zul'atfi Rahmat, Shinsmon Jose, Rajesh Ramasamy, Sharmili Vidyadaran

**Affiliations:** 1 Neuroinflammation Group, Immunology Laboratory, Department of Pathology, Faculty of Medicine and Health Sciences, Universiti Putra Malaysia, 43400 Serdang, Malaysia; 2 Stem Cells & Immunity Group, Immunology Laboratory, Department of Pathology, Faculty of Medicine and Health Sciences, Universiti Putra Malaysia, 43400 Serdang, Malaysia

## Abstract

**Introduction:**

Mesenchymal stem cells (MSCs) are immunosuppressive, but we lack an understanding of how these adult stem cells are in turn affected by immune cells and the surrounding tissue environment. As MSCs have stromal functions and exhibit great plasticity, the influence of an inflamed microenvironment on their responses is important to determine. MSCs downregulate microglial inflammatory responses, and here we describe the mutual effects of coculturing mouse bone marrow MSCs with BV2 microglia in a lipopolysaccharide (LPS) inflammatory paradigm.

**Methods:**

Mouse MSCs were cultured from femoral and tibial bone marrow aspirates and characterized. MSCs were cocultured with BV2 microglia at four seeding-density ratios (1:0.2, 1:0.1, 1:0.02, and 1:0.01 (BV2/MSC)), and stimulated with 1 μg/ml LPS. In certain assays, MSCs were separated from BV2 cells with a cell-culture insert to determine the influence of soluble factors on downstream responses. Inflammatory mediators including nitric oxide (NO), interleukin-6 (IL-6), tumor necrosis factor-alpha (TNF-α), and chemokine (C-C motif) ligand 2 (CCL2) were measured in cocultures, and MSC and BV2 chemotactic ability determined by migration assays.

**Results:**

We demonstrated MSCs to increase expression of NO and IL-6 and decrease TNF-α in LPS-treated cocultures. These effects are differentially mediated by soluble factors and cell-to-cell contact. In response to an LPS stimulus, MSCs display distinct behaviors, including expressing IL-6 and very high levels of the chemokine CCL2. Microglia increase their migration almost fourfold in the presence of LPS, and interestingly, MSCs provide an equal impetus for microglia locomotion. MSCs do not migrate toward LPS but migrate toward microglia, with their chemotaxis increasing when microglia are activated. Similarly, MSCs do not produce NO when exposed to LPS, but secrete large amounts when exposed to soluble factors from activated microglia. This demonstrates that certain phenotypic changes of MSCs are governed by inflammatory microglia, and not by the inflammatory stimulus. Nonetheless, LPS appears to "prime" the NO-secretory effects of MSCs, as prior treatment with LPS triggers a bigger NO response from MSCs after exposure to microglial soluble factors.

**Conclusions:**

These effects demonstrate the multifaceted and reciprocal interactions of MSCs and microglia within an inflammatory milieu.

## Introduction

Mesenchymal stem cells (MSCs) regulate a wide range of immune cells [1,2]. They limit proliferation of T and B lymphocytes [3-5], prevent differentiation of monocytes into dendritic cells [6,7], and inhibit dendritic cell maturation [8]. During tissue injury, inhibitory functions of MSCs appear to be elicited by inflammation, with the requirement of MSC "licensing" by inflammatory mediators shown to be necessary for their subsequent immunosuppressive activities [9-11].

A role for MSCs in ameliorating disease within the central nervous system (CNS) is being defined. In animal models of stroke [12] and Alzheimer's disease [13], MSCs appear to improve disease by dampening localized inflammatory responses. Other therapeutic features of MSCs, such as their regenerative and (trans)differentiation abilities, seem to have less to do with alleviating the pathology of CNS diseases [14,15]. The resident inflammatory cells of the CNS are microglia [16]. Derived from primitive myeloid progenitors that form a pool of resident microglia in the adult brain [17], these CNS-specific macrophages initiate and maintain inflammatory responses in the brain and spinal cord. In response to infection or injury, microglia assume a proinflammatory phenotype, secreting various mediators, including cytokines, reactive oxygen and nitrogen species, chemokines, and neurotrophic factors [16]. Several lines of evidence attribute neuronal damage to the inflammatory responses of microglia, and not necessarily to a direct neurotoxic insult [18-20]. Accordingly, reactive microglia are a common feature of numerous brain pathologies [21], and improvement of disease outcome with MSC transplantation is accompanied by modulation of microglia proliferation [13,22], inflammatory mediators [23,24], and phagocytosis [13,15].

The inflammatory responses of the brain and spinal cord are unique, initially restricted to reactions of microglia and astrocytes, not circulating systemic leukocytes [25,26]. Unlike other tissue macrophages, microglia proliferate and appear to have limited antigen-presenting capability [16]. These distinct properties of microglia, coupled with the fact that MSCs are highly responsive to their environment [27], warrant the need to determine their mutual interactions during neuroinflammation. To understand the mechanism of MSC-mediated downregulation of neuroinflammation, it is important to examine the influence of the inflamed environment on MSCs. As multipotent cells with trophic functions, MSCs are highly responsive to biologic cues within their locality. Understanding how neuroinflammation affects MSCs is crucial to gauge their long-term therapeutic efficacy. Reports describing an immune response against MSCs transplanted into the brain underscore the need to understand endogenous, organ-specific cellular reactions toward MSCs [28,29]. Here, we determined nitric oxide (NO) secretion, inflammatory cytokine expression, and chemotactic activity of both cell types in response to a lipopolysaccharide (LPS) stimulus. We now appreciate that great reciprocities exist between the two cells and that MSCs react to signals from both microglia and the inflammatory agent, with cues from both affecting MSCs in distinct ways.

## Materials and methods

### Ethics statement

Use and care of animals were approved by the Animal Care and Use Committee (ACUC, Approval ID UPM/FPSK/PADS/BRUUH/00163), Faculty of Medicine and Health Sciences, Universiti Putra Malaysia.

### Mouse bone marrow cell culture

MSCs were generated from bone marrow of the tibias and femurs of male BALB/c and ICR mice. Cells were cultured in Dulbecco modified Eagle medium (DMEM) with high glucose concentration, 10 n*M *GlutaMAX-I Supplement, 100 U/ml penicillin, 100 μg/ml streptomycin, and 10 μg/ml gentamycin (all from Invitrogen, CA, USA) and supplemented with 15% Mesenchymal Stem Cell Stimulatory Supplements (STEM-CELL Technologies, Canada). Culture of the cells was based on a modified protocol [30]. In brief, 3 hours after culture, media was replaced, followed by subsequent media changes and 1× PBS washes every 6 hours for 72 hours. Cells were harvested by treating with 0.25% trypsin containing 1 m*M *EDTA for 5 minutes at 37°C. MSC cultures were characterized by positive expression for CD106, Sca-1, and CD44, and negative expression for CD45, CD11b, and MHC class II (all from BD Biosciences, San Jose, CA, USA). Differentiation assays were also performed by using the Millipore Mesenchymal Stem Cell Adipogenesis Kit (SCR020, Merck KGaA, Darmstadt, Germany) and Mesenchymal Stem Cell Osteogenesis Kit (SCR028, Merck KGaA, Darmstadt, Germany). Cells at passages eight through 14 were used for downstream experiments.

## BV2 microglia culture

BV2 cells were a generous gift from Dr. Thameem Dheen of the National University Singapore. BV2 is an immortalized microglia cell line infected with a v-*raf*/v-*myc *oncogene carrying retrovirus J2 [31]. Cells were cultured in DMEM with 5% heat-inactivated fetal bovine serum (FBS), 100 U/ml penicillin, 100 μg/ml streptomycin, 10 μg/ml gentamycin (all from Invitrogen, CA, USA), 1× nonessential amino acids, and 6.25 μg/ml insulin (Sigma-Aldrich, St. Louis, MO, USA). Cells were harvested on reaching 80% to 90% confluence by treating with 0.25% trypsin containing 1 m*M *EDTA for 5 minutes at 37°C.

### MSC/BV2 cocultures

MSC and BV2 cells were cocultured at four different ratios of BV2 cells to MSCs, 1:0.2, 1:0.1, 1:0.02, and 1:0.01, with the BV2 cell number kept constant. Cells were seeded simultaneously (except for proliferation assays) and incubated overnight at 37°C in a 5% CO_2 _incubator to allow cells to adhere. Cocultures were then stimulated with 1 μg/ml lipopolysaccharide (LPS; *Escherichia coli *serotype O26:B6; L2762 Sigma-Aldrich, St. Louis, MO, USA), and the time point of LPS addition considered as 0 hours for all experiments. Cell-culture inserts with a 1-μm polyethylene terephthalate membrane pore size (BD Falcon; BD Biosciences, Belgium) were used for transwell experiments.

### BV2 proliferation

BV2 cell proliferation was determined by assessing tritiated thymidine (^3^H-TdR; Perkin Elmer Boston, USA) incorporation. In 96-well plates, MSCs were seeded in triplicate at densities corresponding to the coculture ratios mentioned earlier and allowed to adhere overnight. The following day, MSCs were treated with 10 μg/ml mitomycin-C (Sigma-Aldrich, St. Louis, MO, USA) for 2 hours to halt their proliferation. Plates were then washed thoroughly with DMEM to remove any traces of the mitotic inhibitor, and BV2 cells were then seeded at 5 × 10^3 ^BV2 cells/well. Cocultures were activated with 1 μg/ml LPS for 48 hours, and ^3^H-TdR (0.037 MBq/well (0.5 μCi/well)) was added to wells at the final 6 hours of incubation. Plates were then exposed to a freeze/thaw cycle at -20°C to ease cell harvesting. Cells were harvested onto a filter mat by using an automated cell harvester (Harvester Mach III M; TOMTEC, CT, USA). Thymidine incorporation was measured with liquid scintillation spectroscopy on a beta counter (MicroBeta^® ^TriLux; Perkin Elmer, USA) after the addition of scintillation fluid (OptiPhase SuperMix Cocktail; Perkin Elmer Boston, USA), and readouts were in counts per minute (cpm).

### Detection of NO and cytokines

In 12-well plates, BV2 cells (2 × 10^5 ^cells/well) were cocultured overnight with the varying MSC ratios, with and without transwell, and stimulated with 1 μg/ml LPS the following day. NO was detected in the supernatant of cocultures by using the Griess assay. For this, 50 μl of culture supernatant from each sample was transferred to a 96-well plate in triplicate and added with the same volume of Griess reagent (1% sulfanilamide/0.1% *N*-1-napthylethylenediamine dihydrochloride/2.5% phosphoric acid; all from Sigma-Aldrich, St. Louis, MO, USA). Absorbance was read at 530 nm (MRX II microplate reader, Dynex Technologies, VA, USA) after 10-minute incubation. Nitrite concentration was calculated with reference to a standard curve of freshly prepared sodium nitrite (0 to 100 μ*M*). For supernatant transfer studies, 2 × 10^5 ^BV2 cells/well were cultured in 12-well plates for 24 hours, with or without LPS, and the supernatants collected and analyzed for NO to determine basal NO levels. The supernatants were transferred to MSC cultures in 12-well plates (at the ratios described earlier), completely replacing MSC culture medium. NO levels were then analyzed at 48, 72, and 96 hours. The basal levels of NO_2_^- ^in resting and LPS-activated BV2 cells were subtracted from the absorbance reading from MSC cultures to estimate the contribution by MSCs. The results are displayed as micromolar concentration of NO_2_.

Coculture supernatants were assayed at 6, 24, and 48 hours for IL-6, IL-10, MCP-1 (CCL2), IFN-γ, TNF, and IL-12p70 by using a multiplex bead array kit (BD Cytometric Bead Array mouse inflammation kit; BD Biosciences, San Jose, CA, USA), according to the manufacturer's instructions. Samples were assayed on a FACS Fortessa flow cytometer (BD Biosciences, San Jose, CA, USA) and analyzed with FCAP array software (BD Biosciences, San Jose, CA, USA). Concentration of cytokines in samples was calculated by using individual standard curves and expressed as pictograms per milliliter and fold change.

### Migration assay

BV2 (1 × 10^5 ^cells) or ICR-derived MSCs (2 × 10^4 ^cells) was seeded onto cell-culture inserts with a polyethylene terephthalate membrane pore size of 8 μm (for BV2 cells) and 3 μm (for MSCs) in 24-well plates. At 6 hours (for BV2 cells) and 24 hours (for MSCs), culture media in inserts were carefully removed, and the insert membrane gently wiped with a cotton swab to remove cells that did not migrate across the membrane. The inserts were then rinsed with PBS, cells fixed with 2% paraformaldehyde, permeabilized with 0.01% Triton X-100 (Sigma-Aldrich, St. Louis, MO, USA), and stained with crystal violet (Sigma-Aldrich, St. Louis, MO, USA). Nine fields at 20× magnification were imaged by using phase-contrast microscopy, and the number of cells that migrated across the membrane were counted.

### Statistical analysis

Significance was assessed by using one-way ANOVA followed by the Tukey *post hoc *test or Student *t *test by using GraphPad Prism version 6 (GraphPad Software, CA, USA).

## Results

### MSCs inhibit BV2 proliferation

MSCs derived from BALB/c mouse bone marrow were CD106^+^Sca-1^+^CD44^high^MHC I^-^CD45^-^CD11b^-^MHC II^-^. The cells demonstrated their multipotentiality by differentiating into adipocytes and osteocytes (see Additional file 1). MSCs derived from ICR mouse bone marrow were previously characterized by our group [32].

**Figure 1:**
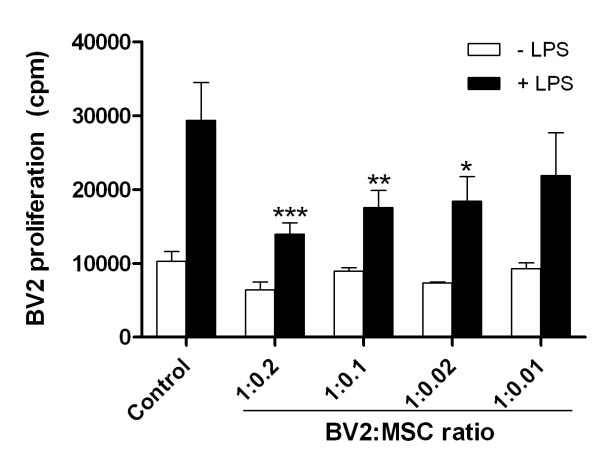
**MSCs inhibit BV2 microglia proliferation**. MSCs and BV2 microglia were cocultured at ratios indicated below the graph, and BV2 proliferation was determined with ^3^H-TdR incorporation 48 hours after stimulation with 1 μg/ml LPS. Values are expressed as mean ± SD and from a representative of two independent experiments. *P < 0.05; **P < 0.01; ***P < 0.001, compared with respective controls. cpm, counts per minute; ^3^H-TdR, tritiated thymidine; LPS, lipopolysaccharide; MSCs, mesenchymal stem cells; SD, standard deviation.

LPS increased BV2 microglia proliferation by close to threefold (Figure 1). Coculturing BV2 with MSC decreased BV2 proliferation in a dose-dependent manner. At 0.2, 0.1, and 0.02 seeding-density ratios, inhibition of LPS-stimulated BV2 microglia proliferation by MSCs was significant (Figure 1). Inhibition was best at the highest coculture ratio of 1:0.2 (BV2: MSCs), reducing LPS-stimulated BV2 proliferation by 52% (*P *< 0.001). It is unlikely that this decrease in proliferation is due to a lack of nutrients in coculture, as MSCs were mitotically arrested with mitomycin-C, and the culture media pH indicator remained pink/red. Seeding densities of cocultures were also optimized to ensure sufficient room for cell growth in culture plates. MSCs did not significantly inhibit proliferation of unstimulated microglia.

### MSCs increase NO production in BV2 cocultures

Microglia are robust producers of nitric oxide (NO), and the Griess assay was performed to determine the effect of MSCs on NO production in MSC/BV2 cocultures. BV2 and MSCs were cocultured in 12-well plates at the varying ratios and stimulated with LPS. Inactivated BV2 cells produce negligible NO (see Additional file 2). MSCs do not express NO, regardless of LPS stimulation (Additional file 2). To determine whether the presence of MSCs alone affects NO production, BV2 cells were cocultured with MSCs at the highest ratio (1:0.2) without LPS. In these cocultures, NO levels were also insignificant (Additional file 2).

**Figure 2:**
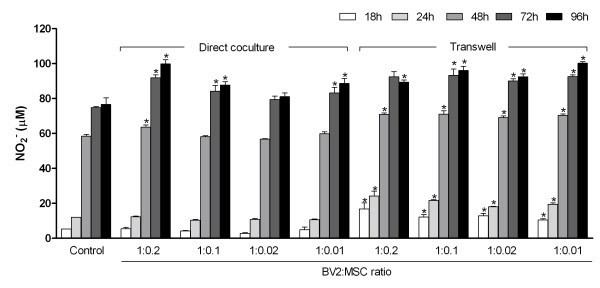
**MSCs increase NO production in MSC/BV2 cocultures**. MSCs and BV2 cells were seeded simultaneously into 24-well plates at ratios indicated below the graph. After overnight incubation, 1 μg/ml LPS was added to cocultures, and NO_2_^- ^concentration was determined with the Griess assay at 18, 24, 48, 72, and 96 hours. Values are expressed as mean ± SD of triplicate wells and from a representative of two independent experiments. Control is BV2 cells treated with LPS. *P < 0.05 compared with control for direct cocultures and compared with direct coculture for transwell cocultures. LPS, lipopolysaccharide; MSC, mesenchymal stem cell; NO, nitric oxide; NO_2_^-^, nitrite; SD, standard deviation.

Activating BV2 cells with 1 μg/ml LPS increased NO production up to 76.64 ± 3.76 μ*M *at 96 hours (Figure 2). Increased NO was detected when BV2 cells were cocultured with MSCs in the presence of LPS. The highest coculture ratio of 1:0.2 increased NO levels by 5.17 ± 1.19 μ*M *at 48 hours, 17.07 ± 1.71 μ*M *at 72 hours, and 23.21 ± 2.41 μ*M *at 96 hours (*P *< 0.05) compared with LPS-stimulated BV2 alone. Cocultures of 1:0.1 ratio showed a moderate increase in NO production by 9.31 ± 3.41 μ*M *at 72 hours and 11.11 ± 1.92 μ*M *at 96 hours (*P *< 0.05). Coculture ratio 1:0.02 showed no considerable difference in NO levels compared with control, whereas the 1:0.01 ratio showed a modest increase of 8.44 ± 3.17 μ*M *and 11.98 ± 2.88 μ*M *at 72 and 96 hours, respectively (*P *< 0.05).

### Cell-to-cell contact is not crucial for NO increase in cocultures

To determine the role for cell-to-cell contact in the increase of NO production in MSC/BV2 cocultures, experiments were performed with MSCs separated from BV2 cells in culture wells by transwell inserts of 1-μm pore size. As with direct cocultures, NO levels in transwell cocultures remained higher than control for all ratios (Figure 2). Compared with that in direct cocultures, NO expression in transwell cocultures was higher at 18 and 24 hours for all ratios (*P *< 0.05). NO levels in the 1:0.2 ratio of direct cocultures at 96 hours was reduced in transwell cocultures (99.85 ± 1.39 μ*M *compared with 89.36 ± 0.75 μ*M*; *P *< 0.05); however, it remained higher than control. For other MSC/BV2 ratios, NO was increased in transwell cocultures, by 9 to13 μ*M*, compared with direct cocultures (*P *< 0.05).

### Soluble factors from activated microglia trigger MSCs to produce NO

The increased levels of NO in cocultures may be attributable to MSCs producing their own NO. Although LPS alone does not trigger MSCs to secrete NO (Additional file 2), it remains possible that in the presence of microglia, MSCs are able to generate NO. To investigate this, we assessed NO production by MSCs after addition of supernatant from BV2 cultures. For this, MSCs and BV2 were cultured separately, and after 24 hours, BV2 supernatant (with or without LPS activation) was transferred onto MSC cultures.

**Figure 3:**
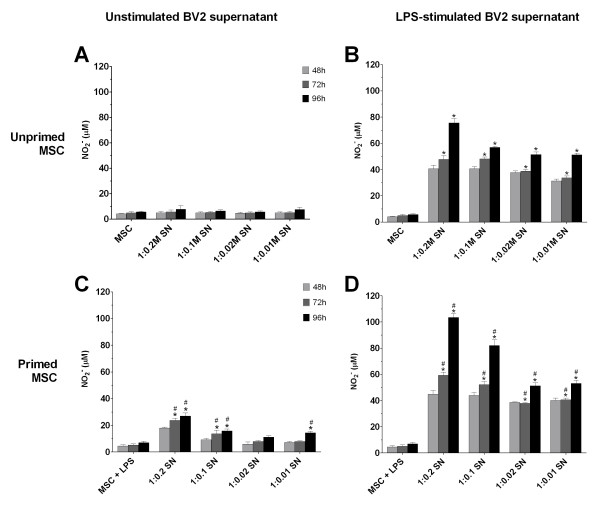
**MSC production of NO is dependent on soluble factors from LPS-stimulated BV2 cells, and increases with LPS priming**. Unprimed **(A, B) **and LPS-primed **(C, D) **MSCs were seeded at densities corresponding to coculture ratios and treated with supernatant (SN) from unstimulated (A, C) and LPS-stimulated (B, D) BV2 cultures. Values are expressed as mean ± SD of triplicate wells and from a representative of two independent experiments. *P < 0.05 compared with respective MSC controls (for D, values were compared with corresponding values in C). ^#^*P *< 0.05 compared with corresponding values from unprimed MSCs. LPS, lipopolysaccharide; MSC, mesenchymal stem cell; NO, nitric oxide; SD, standard deviation.

The production of NO by MSCs after addition of unstimulated BV2 supernatant was insignificant at all seeding ratios tested (Figure 3A). LPS-activated BV2 supernatant, however, stimulates MSCs to produce NO. At the highest coculture ratio of 1:0.2, MSCs produced up to 75.51 ± 3.27 μ*M *NO at 96 hours (*P *< 0.05; Figure 3B). Even as MSC number was reduced to 2 × 10^3 ^(at the 1:0.01 seeding ratio), NO production remained high at 51.31 ± 1.19 μ*M *at 96 hours (*P *< 0.05).

### Priming MSCs with LPS amplifies subsequent NO production

Experiments were also performed with MSCs treated with 1 μg/ml LPS for 24 hours (termed primed MSCs hereafter) before addition of unstimulated or LPS-stimulated BV2 culture supernatant. This was to determine whether rendering MSCs into an inflammatory state primes them to behave differently when later exposed to soluble factors of BV2 microglia.

Priming MSCs with LPS did not stimulate NO production (Figure 3C; MSC + LPS). Interestingly, addition of unstimulated BV2 supernatant significantly increases NO secretion by primed MSCs (with MSC number corresponding to the 1:0.2 ratio) to 26.86 ± 2.37 μ*M *at 96 hours (*P *< 0.05; Figure 3C). At the seeding-density ratio of 0.01, NO levels remained significantly higher than primed MSCs alone at 14.40 ± 1.22 μ*M *at 96 hours (*P *< 0.05). Exposing primed MSCs to LPS-stimulated BV2 supernatant further increased NO production (Figure 3D). For instance, NO levels in the 1:0.2 ratio of primed MSCs exposed to LPS-activated BV2 supernatant surged to 103.41 ± 3.22 μ*M *at 96 hours, significantly higher compared with 75.51 ± 3.27 μ*M *NO produced by unprimed MSCs exposed to LPS-stimulated BV2 supernatant (*P *< 0.05; Figure 3B).

### MSCs increase IL-6 and decrease TNF-*α *expression in cocultures

To determine the inflammatory cytokines possibly modulated by MSCs, we subjected the supernatant of cocultures to a cytokine protein array for IL-6, IL-10, CCL2, IFN-γ, TNF, and IL-12p70. Production of IL-10, IFN-γ, and IL-12p70 was unremarkable, whether for BV2 and MSCs alone or for cocultures (data not shown). Unremarkable expression of IL-10 and IL-12 cytokines indicates that the suppressive effects of MSCs we describe here are not attributable to these antiinflammatory cytokines.

**Figure 4:**
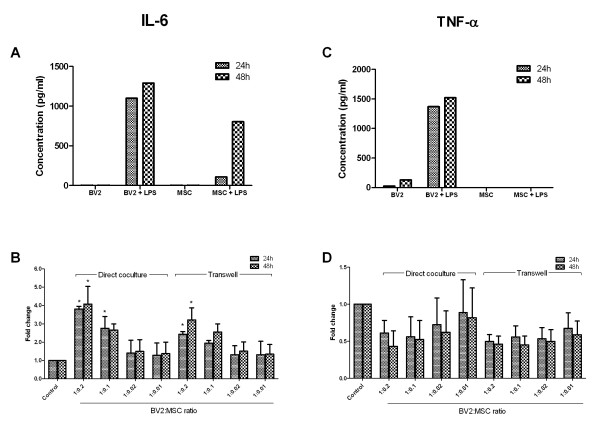
**IL-6 increases and TNF-α decreases in MSC/BV2 cocultures**. **A, C: **IL-6 and TNF-α levels in culture supernatants of BV2 (resting and activated) and MSCs (unprimed and primed) were measured at 24 and 48 hours by using the BD Cytometric Bead Array. Values are expressed in concentration (pg/ml) and from a representative of three independent experiments. **B, D: **BV2 and MSCs were cocultured at ratios indicated below the graph, with or without transwell inserts. Lipopolysaccharide (LPS; 1 μg/ml) was then added to cocultures, and supernatants assayed at 24 and 48 hours by using the BD Cytometric Bead Array. Controls are LPS-treated BV2 cells. Values are fold change compared with control, defined as 1 arbitrary unit and from three independent experiments. Error bars indicate SD. *P < 0.05 compared with respective controls. MSC, mesenchymal stem cell; SD, standard deviation.

BV2 and MSCs have no constitutive production of IL-6 (Figure 4A). However, exposing BV2 and MSCs to LPS induces IL-6 expression in both cells, with BV2 cells producing 1,098.18 pg/ml as early as 24 hours, and MSCs secreting 803.44 pg/ml at 48 hours (Figure 4A). The presence of MSCs increased IL-6 levels in LPS-treated cocultures in a dose-dependent manner. At the 1:0.2 coculture ratio, IL-6 levels increased to 3.80 ± 0.15-fold at 24 hours and 4.08 ± 0.96-fold at 48 hours (*P *< 0.05; Figure 4B). The lower coculture ratios only showed a marginal increase of IL-6; 2.75 ± 0.65 at 24 hours and 2.65 ± 0.34-fold at 48 hours for the 1:0.1 coculture ratio. This increased expression is partially attributable to cell-to-cell contact, as transwell cocultures had reduced IL-6 levels compared with direct cultures, but were higher than control (Figure 4B).

Stimulating BV2 with LPS greatly increased TNF-α expression (1,519.09 pg/ml at 48 hours; Figure 4C). MSCs do not express TNF-α, regardless of LPS treatment (Figure 4C). Converse to IL-6, expression of TNF-α was reduced by MSC dose-dependently in cocultures; to 0.61 ± 0.20 and 0.43 ± 0.20 at 24 and 48 hours for the 1:0.2 ratio (Figure 4D). Downregulation of TNF-α was lost at the lowest 1:0.01 coculture ratio, with levels similar to those of control cultures. Interestingly, separating MSCs from BV2 cells in transwell cocultures appears to confer TNF-α reduction for all coculture ratios, plateauing TNF-α expression to approximately 0.4-0.7-fold of control (Figure 4D). This indicates that a cell-to-cell contact mechanism is responsible for the reduced suppression of TNF-α at coculture ratios 1:0.1, 1:0.02, and 1:0.01, as soluble factors alone reduce TNF-α for these coculture ratios.

### MSCs and microglia migrate reciprocally and express CCL2

Both microglia and MSCs are migratory cells; microglia accumulate around areas of injury in the CNS and MSCs home to sites of tissue injury. By exploring their migratory behaviors, we can determine whether MSCs also immonumodulate microglia responses by limiting their migration toward inflammatory stimuli. Alternatively, we can also establish whether MSCs preferentially move in near vicinity with microglia to exert their modulatory effects.

**Figure 5:**
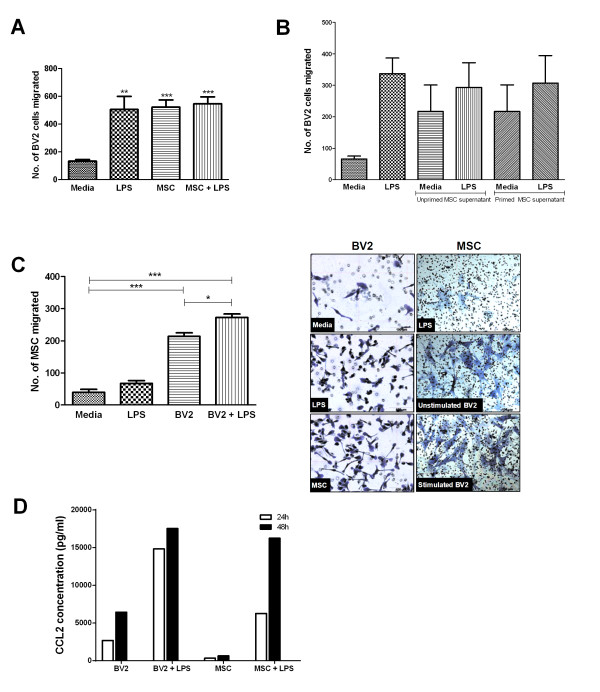
**BV2 and MSCs are migratory toward each other**. Migration was assessed by counting the number of cells stained with crystal violet on the converse side of transwell culture inserts. **(A) **Migration for BV2 microglia was assessed at 6 hours. Values are expressed as mean cell number ± SD and from three independent experiments. **P < 0.01, ***P < 0.001 compared with control. **(B) **Supernatants from 24-hour unprimed and primed MSCs were transferred to BV2 cultures to assess BV2 migration at 6 hours. Values are expressed as mean cell number ± SD and from four independent experiments. **(C) **Migration for MSCs was assessed at 24 hours. Values are expressed as mean cell number ± SD and from three independent experiments. *P < 0.05, ***P < 0.001 compared with control. **(D) **CCL2 levels were assayed at 24 and 48 hours in culture supernatant with the BD Cytometric Bead Array. Values are expressed as mean and from a representative of three independent experiments. CCL2, chemokine (C-C motif) ligand 2; MSC, mesenchymal stem cell; SD, standard deviation.

Unstimulated BV2 cells are migratory, and increase their migration 3.8-fold as early as 6 hours after LPS stimulation (*P *< 0.01; Figure 5A). Interestingly, BV2 cells migrate equally well toward MSCs, and BV2 migration is not affected when cultured with both MSCs and LPS (Figure 5A; MSC + LPS). As BV2 is chemoattracted to MSCs within transwell cocultures, we next determined whether MSCs that are not in contact with microglia can affect microglia chemotaxis via secretion of soluble factors. For this, the supernatants of 24-hour MSC cultures were added to BV2 cultures, and the cells were examined for migration at 6 hours. Unlike coculture experiments, these MSCs do not receive signals from BV2 microglia. We found BV2 migration not significantly affected by soluble factors secreted by unprimed or LPS-primed MSCs (Figure 5B).

MSC, conversely, do not migrate significantly in the presence of LPS (Figure 5C). However, their migratory capacity increases significantly toward BV2 cells (*P *< 0.001), more so if the cultures are exposed to LPS (*P *< 0.001). Both cell types express large amounts of the chemokine CCL2 (MCP-1). For BV2 cells, CCL2 expression increased more than fivefold at 24 hours (to 14,827.30 pg/ml) and almost threefold at 48 hours (to 17,518.00 pg/ml) after LPS stimulation (Figure 5D). MSCs increased CCL2 expression by more than 18-fold at 24 hours (6,268.41 pg/ml) and 25-fold at 48 hours (16,240.90 pg/ml). Coculture of BV2 with MSCs in the presence of LPS did not noticeably affect CCL2 levels (see Additional file 3).

## Discussion

MSCs are ideal cellular candidates for therapy. They expand well in culture, have relatively low tumorigenic and alloreactive risk, and preferentially home to areas of injury [1,2]. MSCs ameliorate disease in various animal models of neuroinflammation [12,13,24,33,34], and these outcomes are attributed mainly to the immunomodulatory properties of MSCs. To ensure their therapeutic efficacy, knowledge of the number of MSCs required to control inflammation, the need for MSCs to be in close vicinity to the site of tissue injury, and the effects of the inflammatory environment on MSCs should be deduced. We demonstrate here that interactions between MSCs and microglia are distinct dependent on cell number and their proximity, and that the inflammatory milieu dictates the modulatory properties of MSCs.

Bone marrow-derived MSCs inhibit proliferation of lipopolysaccharide (LPS)-activated BV2 microglia, signifying the suppressive effects of MSCs on microglia that have previously been reported [32,35]. LPS serves as a trigger for microglia activation to an inflammatory phenotype [23,32,36,37], increasing their proliferation, upregulating expression of activation markers, and inducing production of inflammatory mediators. One of the mediators produced is nitric oxide (NO), generated by inducible NO synthase (iNOS). NO mediates inflammatory responses within the CNS [38-40], and excessive amounts can cause neurotoxicity [19,39].

We demonstrated MSCs to increase NO levels in LPS-treated microglia cocultures. As these cells are grown within the same culture well, we are unable to tag the increase in NO levels in cocultures to BV2 microglia or MSCs; however, we have found both cell types capable of producing their own large amounts of NO. For MSCs, a direct inflammatory stimulus (LPS) does not induce NO production, nor do soluble factors from resting, inactivated microglia. Only when exposed to soluble factors from LPS-activated microglia did we observe MSCs producing substantial amounts of NO. Similarly, MSCs produce NO only when cocultured with stimulated, and not unstimulated, T lymphocytes [41]. Ren and colleagues [10] also showed mouse bone marrow MSCs to secrete high concentrations of NO in a specific paradigm; LPS stimulation fails to induce NO production in MSCs, but exposure to inflammatory cytokines IFN-γ and TNF-α results in an NO surge. Therefore, the biologic cue for MSCs to produce NO seems not to be directly from an inflammatory agent, but rather from the ensuing cellular/tissue reaction. We do, however, show that a preexposure of MSCs to LPS augments NO production by MSCs cultured subsequently with microglial soluble factors. This demonstrates the priming effect of MSCs, appearing as if the inflamed microenvironment prepares and licenses subsequent MSC interactions with microglia within this paradigm. This is corroborated by the fact that both mouse and human MSCs can recognize LPS as they express TLR4 [42], the Toll-like receptor that binds LPS. The pleiotropic functions of NO make it difficult to deduce the implications of the increased NO in MSC/microglia cocultures. T-lymphocyte immunosuppression by MSCs is mediated by NO; MSCs secrete high levels of NO that suppress T-cell proliferation, and inhibition of iNOS restores proliferation of splenocytes in MSC cocultures [10]. In the MSC/microglia model, reducing NO levels by 40% to 50% with an iNOS inhibitor did not affect microglia proliferation [32], and a role for NO in the MSC/microglia paradigm remains undefined.

We also showed distinct responses of MSCs and microglia in terms of inflammatory cytokine secretion. LPS triggers both BV2 microglia and MSCs to produce IL-6. Similarly, ligation of TLR4 has been shown to induce MSCs to secrete IL-6 [42-44]. MSCs also secrete large amounts of IL-6 when exposed to macrophages [45,46] and astrocytes [47]; however, the ensuing effects of IL-6 are unclear. In MSC/microglia cocultures, IL-6 increases in coculture, whereas TNF-α levels decrease in a dose-dependent manner. For T-lymphocyte immunosuppression, IL-6-dependent production of prostaglandin E2 (PGE2) was shown to be important for antiproliferative effects of MSCs [48]. Kim and Hematti [46] suggested that an IL-10-high, IL-12-low, IL-6-high, and TNF-α-low expression pattern defines a subtype of M2 macrophages that may have a role in tissue repair, but cytokine expression for such a tissue-repair subtype is undefined for microglia. The surge in IL-6 was augmented in the presence of both soluble factors and cell-to-cell contact, whereas soluble factors alone were sufficient to reduce TNF-α levels in cocultures. Therefore, if the combinatory effects of an IL-6 surge and a TNF-α reduction are essential to confer microglia modulation, MSCs may require being in the immediate vicinity of activated microglia. Although IFN-γ is strongly implicated in modulating T-cell inhibition by MSCs [10], its levels in MSC/microglia cocultures were negligible.

An interesting question to ask is whether MSCs are required to be in close vicinity to microglia to dampen microglia inflammatory responses. We have already discussed that the reduction in TNF-α does not require cell-to-cell contact between MSCs and microglia. Increased NO in MSC/microglia cocultures also occurs without cell-to-cell contact. These effects appear to be conferred without MSCs having to be in direct contact with microglia. We showed that MSCs do not home toward LPS, but are attracted instead to resting and, more so, to activated BV2 microglia. Similarly, MSCs migrate toward human macrophages, with the authors identifying CCL2, CCL5, and IL-8 as chemotactic signals for MSCs [45]. It is often shown that MSCs home toward areas of tissue damage [49-52], and our results indicate that, within the brain, it is not the inflammatory agent that serves as a homing signal, but mediators secreted by microglia. It would be interesting to determine the chemotactic signals that microglia produce to attract MSCs. Although CCL2 is chemoattractant for MSCs [45,53], we speculate that it does not have an autocrine effect on MSCs, as MSCs do not migrate significantly toward LPS, although it produces large amounts of CCL2.

Microglia themselves actively (and equally) migrate toward both an LPS inflammatory stimuli and MSCs, regardless of the primed status of the stem cells. This is different from that for T lymphocytes that only migrate toward MSCs that have been exposed to proinflammatory cytokines [10]. It appears that microglia are compelled to be in close proximity to MSCs. Although LPS-primed MSCs produced high levels of CCL2, a macrophage chemokine, unprimed MSCs conversely do not secrete CCL2; therefore, the chemoattractant responsible for the pronounced migration of microglia toward unprimed MSCs must be other than CCL2. It is possible that MSCs may be diverting the migration of microglia toward them and away from an inflamed site. We showed that, even without interacting with microglia, MSCs produce soluble factors that increase microglia migration. Perhaps the chemotactic impetus from MSCs encourages microglia to form closer contact with MSCs within the inflamed area for subsequent immunosuppression. These are attractive possibilities, best answered with *in vivo *or *ex vivo *approaches. Interestingly when cultured with T lymphocytes, MSCs are inclined to produce large amounts of T cell-associated chemokines, such as CXCL9, CXCL10, and CXCL11 [10], indicating that MSCs may have distinct mechanisms for modulating different immune cells, and their effects are dictated by the cells with which they come in contact.

## Conclusions

The findings here demonstrate the capacity of MSCs to modulate microglial responses. Mechanisms of MSC regulation of microglia responses appear multipronged, with some effects requiring cell contact, and others, not. MSCs are also very responsive to microglia, and an inflammatory stimulus primes these cells to behave in a distinct manner, revealing their remarkable ability to reform, based on their microenvironment. These features of MSCs are favorable for immunomodulatory therapy, as MSCs appear capable of adapting and regulating in a manner specific to the cellular environment in which they localize.

## Abbreviations

LPS: lipopolysaccharide; MSC: mesenchymal stem cell; NO: nitric oxide.

## Competing interests

The authors declare that they have no competing interests.

## Authors' contributions

ZR performed nitric oxide and proliferation experiments; SJ carried out cytokine bead array and migration assays; RR supervised culture of mesenchymal stem cells; SV and RR conceived and designed the study; ZR, SJ, and SV analyzed the data; and SV wrote the manuscript. All authors read and approved the final manuscript.

## Supplementary Material

Additional file 1 **Phenotype and differentiation capacity of mouse bone marrow-derived MSCs**. (A) BALB/c bone marrow cultures immunophenotyped for MSC markers. Values within quadrants indicate percentage positivity of markers for MSCs derived from BALB/c mouse bone marrow at passage 8. (B) MSCs differentiated into adipocytes and osteocytes by using the Millipore Mesenchymal Stem Cell Adipogenesis Kit and Osteogenesis Kit. To observe adipogenesis, cells were stained for triglycerides with Oil Red O. To observe osteogenesis, cells were stained for aggregated calcium deposits with Alizarin Red. MSC, mesenchymal stem cell. Click here for file

Additional file 2 **Negligible nitric oxide (NO) expression in BV2, MSCs, LPS-treated MSCs, and MSC/BV2 cultures**. NO_2_^- ^was assayed at 18, 24, 48, 72, and 96 hours in 24-well plates with the Griess assay. MSC and BV2 seeding density represent the coculture ratio of 1:0.2. Values are expressed as mean ± SD of triplicate wells and from a representative of three independent experiments. NO, nitric oxide; MSC, mesenchymal stem cell; NO_2_^-^, nitrite; SD, standard deviation. Click here for file

Additional file 3 **MSC/BV2 cocultures do not alter CCL2 expression**. BV2 and MSCs were cocultured together or separated by a transwell cell-culture insert at ratios indicated below the graph. LPS (1 μg/ml) was added to cultures, and supernatants assayed at 24 and 48 hours with the BD Cytometric Bead Array. Values are expressed in pg/ml and from a representative of three independent experiments. CCL2, chemokine (C-C motif) ligand 2; LPS, lipopolysaccharide; MSC, mesenchymal stem cell. Click here for file
