# Supplementary figures and images for: Reciprocal interactions of mouse bone marrow-derived mesenchymal stem cells and BV2 microglia after lipopolysaccharide stimulation
Source: Stem Cell Res Ther. 2013 Jan 28;4(1):12. doi: 10.1186/scrt160 (PMC3706938; doi:10.1186/scrt160)

**A**

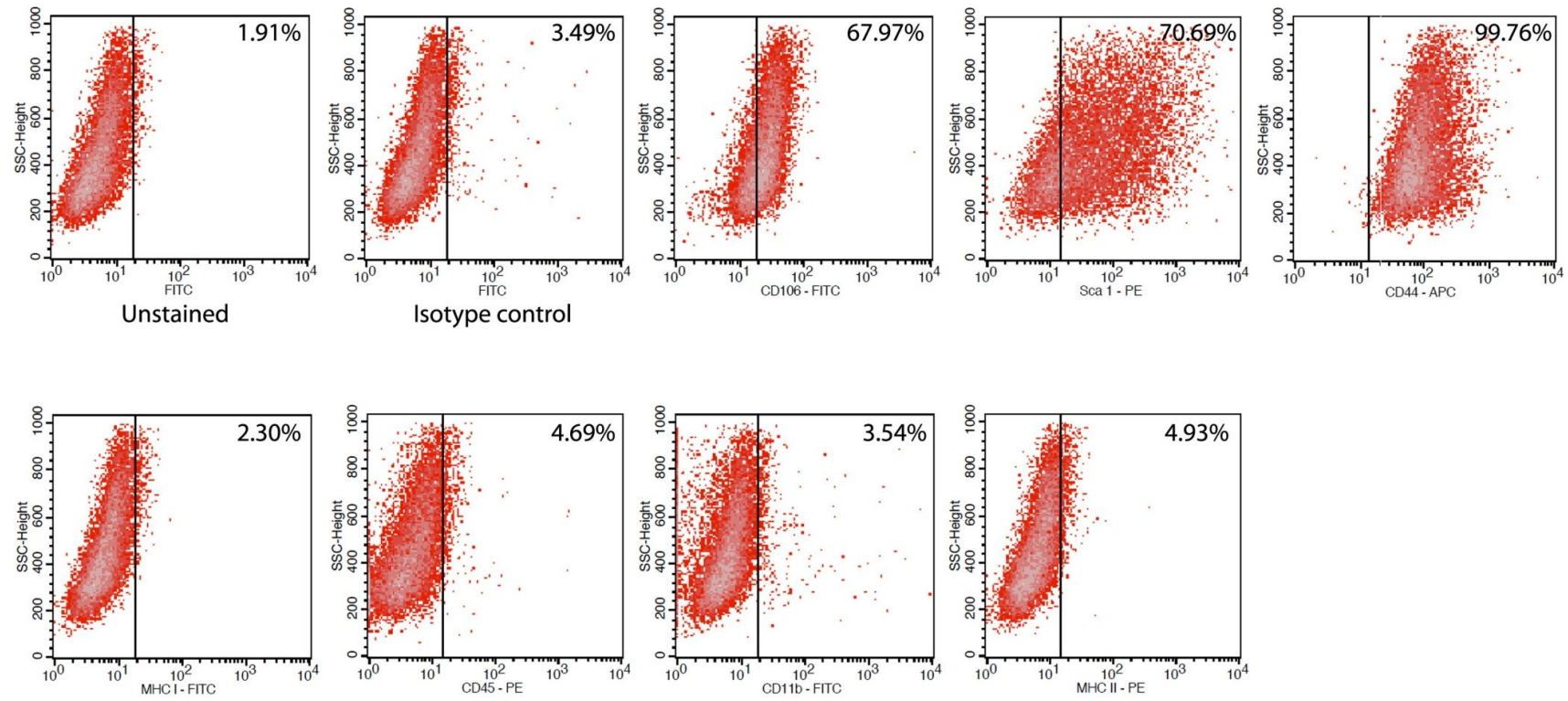

**B**

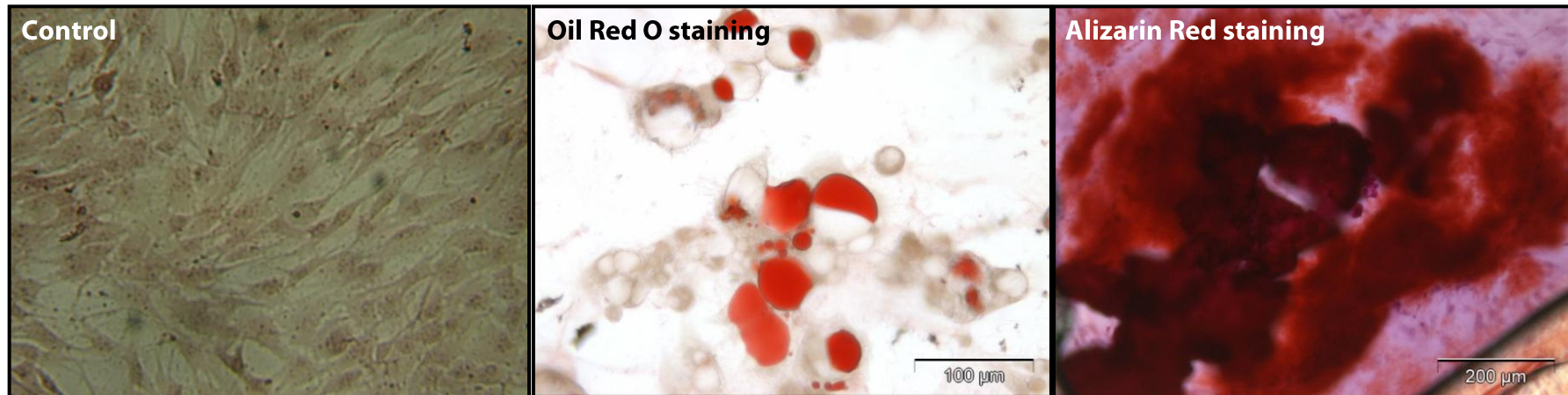

Supplement: Additional file 1 — Phenotype and differentiation capacity of mouse bone marrow-derived MSCs. (A) BALB/c bone marrow cultures immunophenotyped for MSC markers. Values within quadrants indicate percentage positivity of markers for MSCs derived from BALB/c mouse bone marrow at passage 8. (B) MSCs differentiated into adipocytes and osteocytes by using the Millipore Mesenchymal Stem Cell Adipogenesis Kit and Osteogenesis Kit. To observe adipogenesis, cells were stained for triglycerides with Oil Red O. To observe osteogenesis, cells were stained for aggregated calcium deposits with Alizarin Red. MSC, mesenchymal stem cell. [file scrt160-S1.PDF]

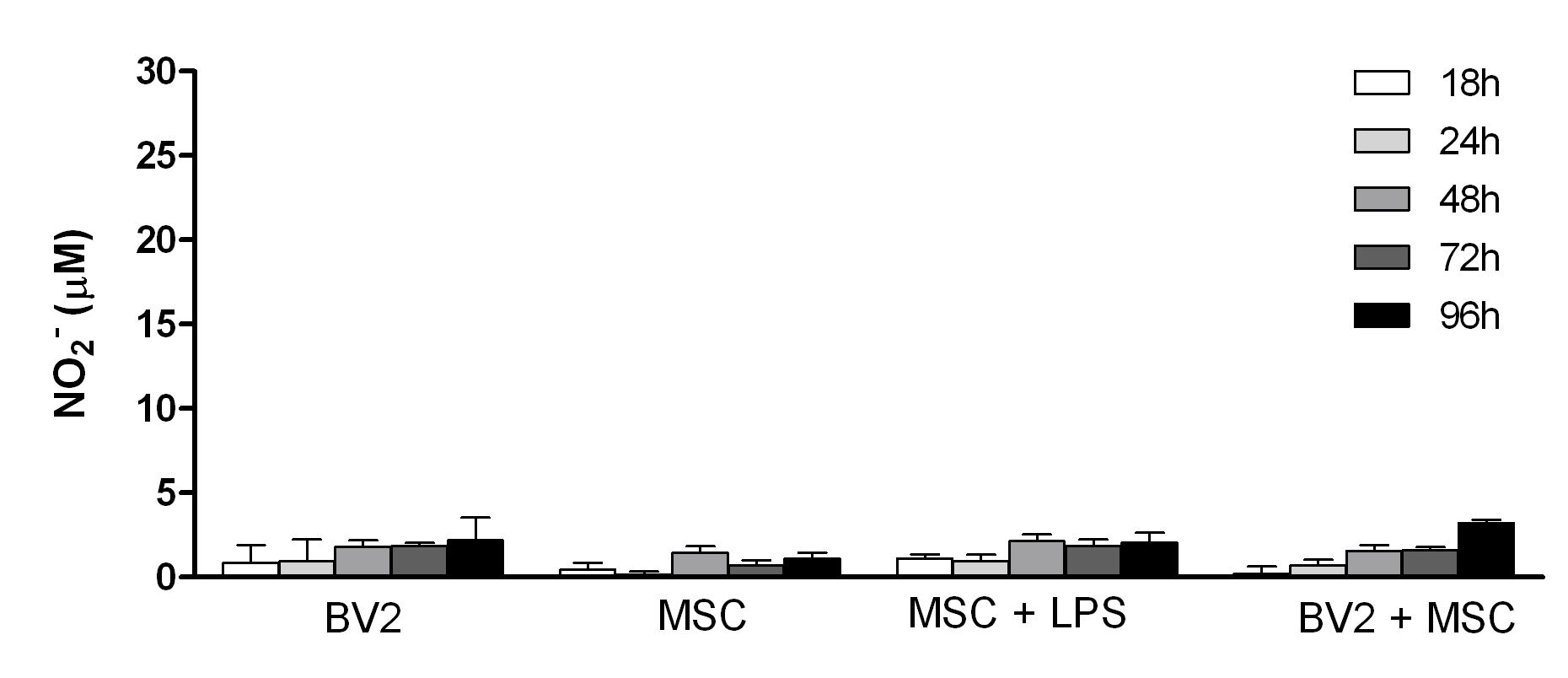

Supplement: Additional file 2 — Negligible nitric oxide (NO) expression in BV2, MSCs, LPS-treated MSCs, and MSC/BV2 cultures. NO2- was assayed at 18, 24, 48, 72, and 96 hours in 24-well plates with the Griess assay. MSC and BV2 seeding density represent the coculture ratio of 1:0.2. Values are expressed as mean ± SD of triplicate wells and from a representative of three independent experiments. NO, nitric oxide; MSC, mesenchymal stem cell; NO2-, nitrite; SD, standard deviation. [file scrt160-S2.TIFF]

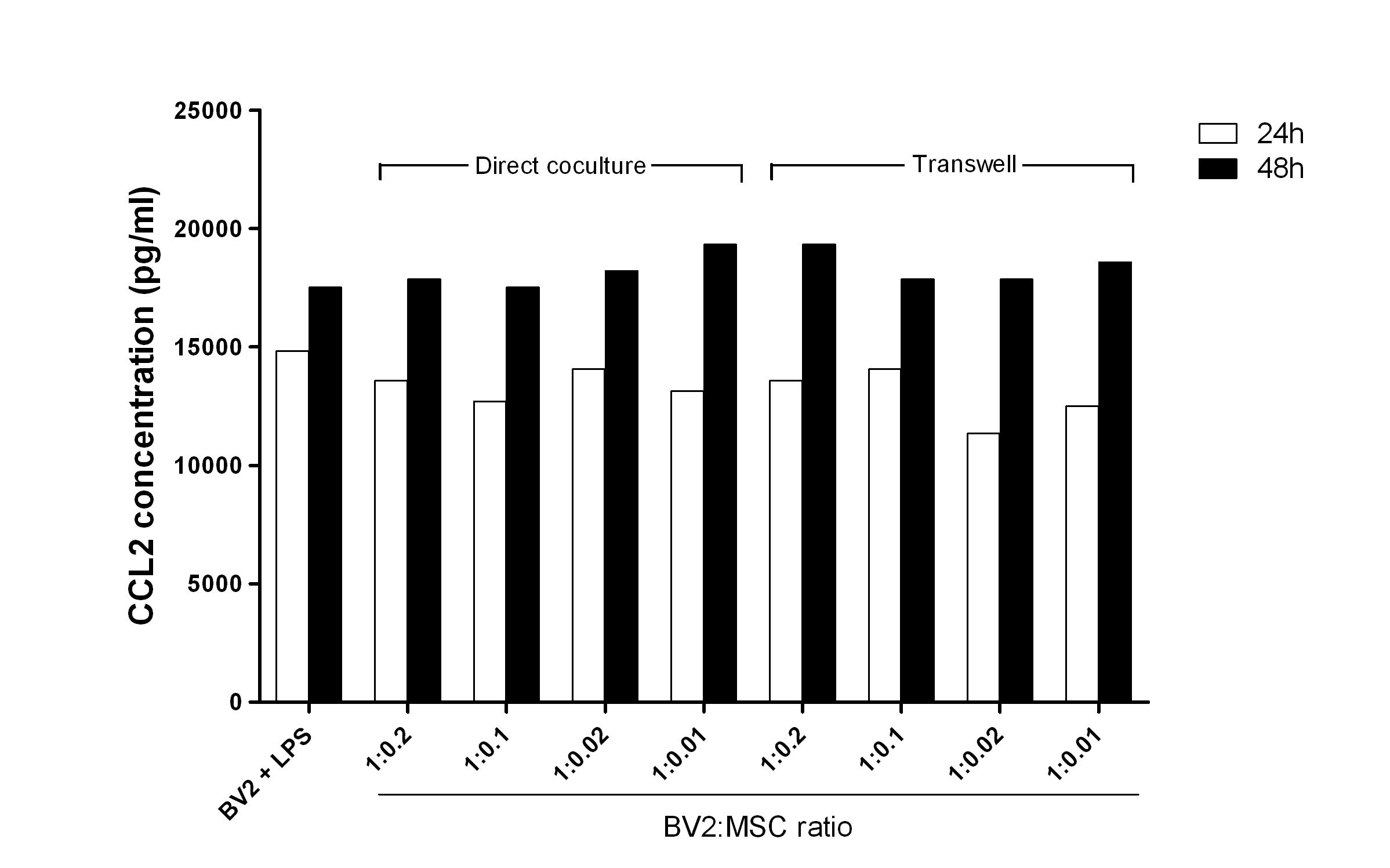

Supplement: Additional file 3 — MSC/BV2 cocultures do not alter CCL2 expression. BV2 and MSCs were cocultured together or separated by a transwell cell-culture insert at ratios indicated below the graph. LPS (1 μg/ml) was added to cultures, and supernatants assayed at 24 and 48 hours with the BD Cytometric Bead Array. Values are expressed in pg/ml and from a representative of three independent experiments. CCL2, chemokine (C-C motif) ligand 2; LPS, lipopolysaccharide; MSC, mesenchymal stem cell. [file scrt160-S3.TIFF]
